# Image‐based shading correction for narrow‐FOV truncated pelvic CBCT with deep convolutional neural networks and transfer learning

**DOI:** 10.1002/mp.15282

**Published:** 2021-10-26

**Authors:** Matteo Rossi, Gabriele Belotti, Chiara Paganelli, Andrea Pella, Amelia Barcellini, Pietro Cerveri, Guido Baroni

**Affiliations:** ^1^ Department of Electronics Information and Bioengineering Politecnico di Milano Milano Italy; ^2^ Bioengineering Unit Clinical Department National Center for Oncological Hadrontherapy (CNAO) Pavia Italy; ^3^ Radiation Oncology Unit Clinical Department National Center for Oncological Hadrontherapy (CNAO) Pavia Italy

**Keywords:** cone beam CT, deep learning, Hounsfield unit recovery, limited FOV, shading correction, transfer learning

## Abstract

**Purpose**: Cone beam computed tomography (CBCT) is a standard solution for in‐room image guidance for radiation therapy. It is used to evaluate and compensate for anatomopathological changes between the dose delivery plan and the fraction delivery day. CBCT is a fast and versatile solution, but it suffers from drawbacks like low contrast and requires proper calibration to derive density values. Although these limitations are even more prominent with in‐room customized CBCT systems, strategies based on deep learning have shown potential in improving image quality. As such, this article presents a method based on a convolutional neural network and a novel two‐step supervised training based on the transfer learning paradigm for shading correction in CBCT volumes with narrow field of view (FOV) acquired with an ad hoc in‐room system.

**Methods**: We designed a U‐Net convolutional neural network, trained on axial slices of corresponding CT/CBCT couples. To improve the generalization capability of the network, we exploited two‐stage learning using two distinct data sets. At first, the network weights were trained using synthetic CBCT scans generated from a public data set, and then only the deepest layers of the network were trained again with real‐world clinical data to fine‐tune the weights. Synthetic data were generated according to real data acquisition parameters. The network takes a single grayscale volume as input and outputs the same volume with corrected shading and improved HU values.

**Results**: Evaluation was carried out with a leave‐one‐out cross‐validation, computed on 18 unique CT/CBCT pairs from six different patients from a real‐world dataset. Comparing original CBCT to CT and improved CBCT to CT, we obtained an average improvement of 6 dB on peak signal‐to‐noise ratio (PSNR), +2% on structural similarity index measure (SSIM). The median interquartile range (IQR) Hounsfield unit (HU) difference between CBCT and CT improved from 161.37 (162.54) HU to 49.41 (66.70) HU. Region of interest (ROI)‐based HU difference was narrowed by 75% in the spongy bone (femoral head), 89% in the bladder, 85% for fat, and 83% for muscle. The improvement in contrast‐to‐noise ratio for these ROIs was about 67%.

**Conclusions**: We demonstrated that shading correction obtaining CT‐compatible data from narrow‐FOV CBCTs acquired with a customized in‐room system is possible. Moreover, the transfer learning approach proved particularly beneficial for such a shading correction approach.

**GLOSSARY**
CBCT: cone beam computed tomography
CNR: contrast‐to‐noise ratio
CT: computed tomography
CTV: clinical target volume
DIR: deformable image registration
D${}_{r}$
: data set containing only real images
D${}_{s}$
: data set containing synthetic CBCT images and real CT
FOV: field of view
FT${}_{x}$
: model trained with transfer learning on x blocks, where x can be 1, 2 or 3
HU: Hounsfield unit
IQR: interquartile range
LOO‐CV: leave‐one‐out cross‐validation
MAE: mean absolute error
noFT: U‐Net model trained without transfer learning
pCT: planning CT
PSNR: peak signal‐to‐noise ratio
ROI: region of interest
SSIM: structural similarity index measure

## 1 INTRODUCTION

Setup correction, efficient target localization, and motion management are the main challenges of accurate radiation therapy. Radiation therapy starts with the acquisition of a 3D planning CT (pCT), which allows the definition of tumor and safety margins along with the dose to be delivered. Weight loss, tumor shrinkage, and air presence in the bowels are well‐known interfractional discrepancies to be accounted for in radiation therapy, especially for tumors located in the pelvic district.^1^ Therefore, the common clinical practice involves using in‐room imaging, with cone beam computed tomography (CBCT) being the most widely adopted technique for anatomical evaluation before fraction delivery in conventional and proton therapies.^2^, ^3^, ^4^, ^5^ Although CBCT is a fast acquiring and versatile solution, it presents limitations with respect to CT modality, such as low contrast and the need to calibrate with respect to CT scanner Hounsfield unit (HU) values.^6^, ^7^ The cone‐shaped beam of CBCT allows much faster imaging compared to a fan beam. Still, it causes a significant amount of scattered radiation, resulting in the infamous cupping artifact in the final image.^8^

The National Center for Oncological Hadrontherapy (CNAO, Pavia, Italy) employs an in‐house developed CBCT system with a nonadjustable field of view (FOV).^2^ This system is used for patient positioning correction (through the acquisition of radiographs) and daily anatomical evaluation before fraction delivery. Although it is not currently employed for daily dose calculation. Instead, CBCT scans from this system are exploited as a tool to trigger an off‐line adaptive procedure based on new CT acquisition and revaluation planning.^9^, ^10^ The main limitations that prevent a proper dose evaluation using the CBCT system installed at CNAO are missing information due to limited FOV and scattering‐induced cupping. At the same time, these limitations hinder the qualitative inspection of the daily anatomy, which leads to suboptimal off‐line procedures and unnecessary imaging dose delivered to the patient. In particular, truncation influences scattering estimation and correction.^11^, ^12^ Limited FOV and truncation artifacts are also not ideal for applying the virtual CT paradigm^3^, ^13^, ^14^, ^15^, ^16^ that aims to recalculate dose on a warped CT to match daily anatomy. The typical approach to map the CT anatomy to that of the daily CBCT usually requires the preprocessing of the CT through deformable image registration (DIR).^17^, ^18^, ^19^ DIR may introduce errors and require extensive validation,^20^ especially when the CBCT volume is flawed and has a narrow FOV. Traditional methods to estimate and correct for scattering in CBCT were extensively described in the literature,^21^, ^22^ being based on prior knowledge of the anatomical target, scattering models to reject the noise and hardware solutions. Literature reveals several precedents in intensity correction using planning CT as a prior. For instance, leveraging the CT information through Monte Carlo (MC) simulations can accurately reproduce CT HU values and allow proton dose calculation.^23^, ^24^ However, the runtime for these corrections requires hours, being incompatible with the clinical setup. Additionally, changes in anatomy between CT and CBCT must be limited, otherwise the CT‐based MC simulation will prove inaccurate for the daily anatomy. More recently, advanced image processing techniques based on neural networks and deep learning were investigated, again leveraging the prior knowledge given by the planning CT. Such networks have demonstrated the ability to integrate information about the targeted anatomical district and negate scattering artifacts.^19^ These methods aimed to correct the CBCT scattering artifacts using the neural encoding–decoding based on the U‐Net CNN architecture, exploiting supervised training. The main requirement for this kind of approach is that of anatomical correspondence between the input image and the ground‐truth reference label. Two main approaches characterize the deep‐learning–based techniques, namely, raw‐data domain scatter correction and image domain shading correction. Regarding raw‐data domain scatter correction, one study proposed to train a U‐Net on CBCT projections corrected with prior information derived from deformed planning CT.^18^ Other works focused instead on training a network by creating MC simulated labels. In a study, the authors trained a deep residual CNN with CBCT scans and corresponding MC scatter‐corrected CBCT scans.^25^ In another proposal, instead, the authors created both the input and the label by synthetic CBCT generation from existing CT and adding MC‐simulated scatter to the input volumes.^11^, ^26^ This was first evaluated on various objects^26^ and then on different anatomical regions^11^ in a reasonable attempt to prove the generality of this method. Finally, the first study using MC‐based CNN CBCT scatter correction on real data was trained on synthetic CBCT scan inputs with added MC‐simulated scatter against unflawed CBCT scans, with particular attention to the scanner trajectory.^27^ Concerning the image domain shading correction, a work involved using DIR between planning CT and CBCT as a preprocessing step followed by a slice‐based supervised training between them.^17^ Slice‐based training is also an emerging trend for the generation of synthetic CT using generative adversarial networks.^28^, ^29^

In order to address the existing image quality limitations at CNAO, the present work adopted the deep learning approach to CBCT axial slice processing by using the U‐Net architecture, exploiting the corresponding CT as a ground‐truth during training.

The clinical data set was retrospectively available at CNAO facility, and it is described in Section 2.2. We enlarged data quantity through a transfer learning approach by generating a synthetic CBCT data set from a publicly available pelvic CT repository,^30^ completely avoiding the use of DIR. It has been shown that pretraining a network with synthetic data could be an effective initialization technique for many complex models, providing better performance when the network is then fine‐tuned with real data.^31^, ^32^ In synthesis, the aim of the present work is to provide a U‐Net–based image domain shading correction for the recovery of CT‐compatible HU values from narrow‐FOV CBCT scans. The main novelty aspect of the present work can be found in the neural network supervised training strategy, which was developed according to the transfer learning paradigm, following an innovative two‐step approach described in Section 2.5. This allowed splitting the learning of the anatomical features from the learning of CBCT/CT shading differences. The performances obtained by the fine‐tuned U‐Net were then compared with a network trained only with the retrospective clinical data set.

## 2 MATERIALS AND METHODS

### 2.1 Imaging instrumentation

**FIGURE 1:**
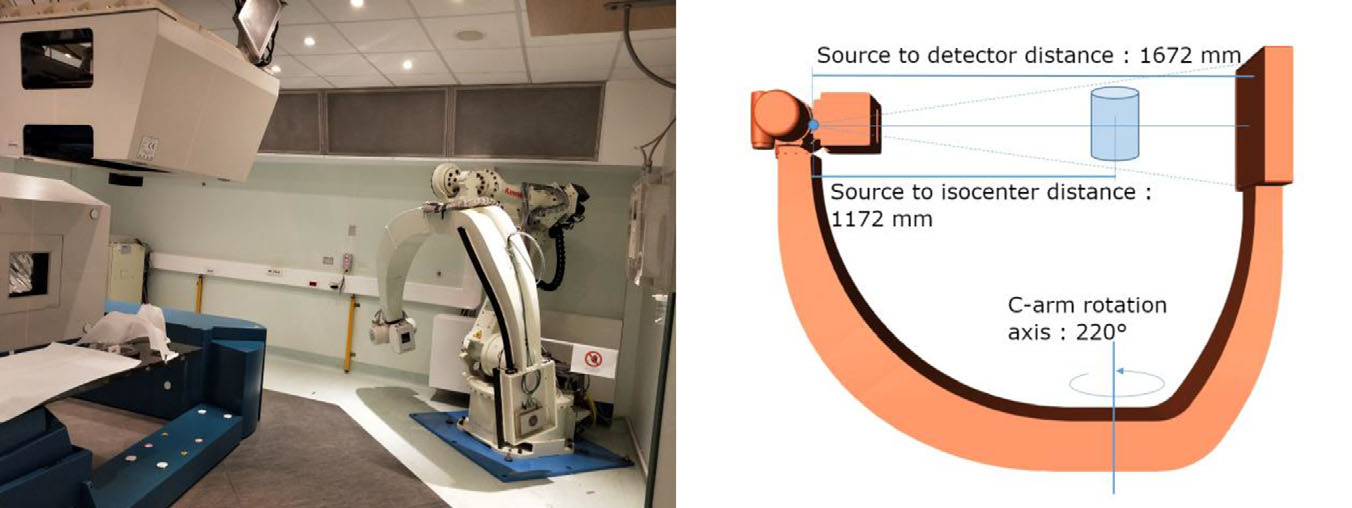
Robotic C‐arm positioned in the treatment room at CNAO. On the couch, it is possible to see a custom thermoplastic fixation mask used during the treatments (left). Schematic representation of the acquisition geometry using the aforementioned C‐arm (right)

In this work, we exploit images from a custom C‐arm solution provided to CNAO particle therapy center^2^ and installed in one of its treatment rooms (Figure 1). It was developed in collaboration with Politecnico di Milano, and it is used for both X‐ray and Full Fan CBCT acquisitions inside the treatment room before the fraction delivery. Planar imaging is exploited for rigid alignment through a 2D to 3D registration framework using planning data (pCT). CBCT is instead acquired for evaluating anatomopathological variations between the planning CT and the daily anatomy. The device was primarily intended for rigid setup correction and is not fitted with a moving flat‐panel or an adjustable collimator. Therefore, it cannot produce a sufficiently wide FOV for a complete anatomical description of larger districts (i.e., pelvis and thorax) via half fan modality. Missing information about the periphery of a large anatomical district does hinder adaptive approaches based on such images. One of the lateral rooms at CNAO is due to receive a similar CBCT system, upgraded with half fan capabilities. Currently, the system already in place is used to evaluate the targeted anatomical district qualitatively. Clinical practice at CNAO reduces inter/intra fractional motion with thermoplastic fixation masks.^33^ However, particle therapy is more susceptible to air cavities with respect to photon radiotherapy. Here a clinical instrument such as the limited FOV CBCT is used to qualitatively evaluate air in the bowels (when dealing with the pelvis). Indeed, the limited FOV of CNAO reconstructed CBCT causes truncation, especially in the case of large anatomical districts such as the pelvis. Consequently, bright‐band effects appear on the border of the FOV along with the nonuniqueness of the interior problem, as stated by Clackdoyle et al.^34^ The issue of truncation artifact correction is attenuated by image processing techniques, such as the Ohnesorge filter,^35^ which produces an undue lowering of the image grayscale intensity. As such, the need for strategies to correct shading due to different components of artifacts is required to improve the qualitative clinical procedure.

### 2.2 Data sets

For the realization of this work, two data sets were used. The first data set, denominated D${}_{r}$, included 18 retrospective pairs of CT/CBCT pelvis acquisitions obtained from six oncological patients (three male/three female). Clinical target volume (CTV) segmentation was also recovered for each patient. More information about the couples is presented in the supporting information (S1). These data were acquired at the CNAO facility during daily pelvic district treatments. The CT scans were acquired with a Siemens Somatom Sensation Open Bore scanner at 100 kV, while the CBCT scans were acquired with a Varian A‐277 X‐ray tube and a Varian PaxScan 4030D flat‐panel detector at 100 kV and 25 mAs, featuring an FOV of 208 mm^2^ and processed by a 30% truncation correction with Ohnesorge filter.^35^ To reduce morphological variations between corresponding CT and CBCT acquisitions, the shortest time interval determined the pairing. In 13 cases, the pairs were acquired on the same day, while four were acquired one day apart. In a single case, the acquisitions were made two days apart. The CT scans are temporally close to the CBCT ones as they are reevaluation CT scans. Given that CNAO employs a thermoplastic mask for patient immobilization and motion reduction,^33^ residual deformation between the corresponding scan was deemed negligible. A more in‐depth analysis is presented in the supporting information (S2), especially regarding DIR on low contrast CBCT scans. Following preprocessing (highlighted in Section 2.3), the data set contained 3368 CT/CBCT 2D axial slices pairs, aligned and rigidly registered between the two modalities. The second data set, termed pelvic reference data,^30^ was obtained from the Cancer Imaging Archive^1^. In this data set, 58 pelvic CT scans, acquired in prone and supine positions, were available. Because of large organ deformation, due to a support cushion placed under the pelvic area, some scans were removed from the useful set. For the remaining 46 patients (27 male/19 female), 8289 CT axial projections were available. The synthetic CBCT images, whose generation was described in Section 2.2.1, joined to the corresponding CT scans, were denominated D${}_{s}$. Both data sets were divided into training, validation, and test sets with a ratio of 60/20/20%. The number of images in every subset was 2021/673/674 for $D_{r}$ and 6632/828/829 for $D_{s}$.

#### 2.2.1 Synthetic data generation

Synthetic CBCT scans were generated from the corresponding CT scans of data set D${}_{s}$ using the OpenREGGUI open‐source platform, written in Matlab and based on the RTK API (the Reconstruction ToolKit^36^). RTK expects intensity values to be in a $0-2^{16}$ range, with an open field signal ($I_{0}$) being on the right‐bound and entirely blocked signal ($I_{dark}$) on the interval's left‐bound. Projections are then log‐transformed to attenuation. In order to obtain a correct projection data set from a CT, the following formula was applied to its grayscale values:

(1) $$CT_{\mu }=\frac{\left(CT_{HU}+1000\right)}{2^{16}}.$$

**FIGURE 2:**
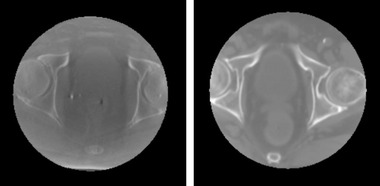
Axial view of a D${}_{r}$ CBCT (left) and a D${}_{s}$ CBCT (right)

The generation of synthetic CBCT was done following the same geometry of CNAO treatment room: source‐to‐detector distance equal to 1672 mm, source‐to‐isocenter distance equal to 1172 mm, and gantry sweep of 220 degrees. A set of 500 simulated cone beam projections was derived, using the RTK CUDA‐based forward projector^36^ with a panel size of 1024 × 768 pixels (spacing 0.388×0.388 mm) for each considered CT. A simulation of scattering and beam hardening was then applied to the projections using the methods provided in OpenREGGUI.^37^ Finally, a CBCT axial scan was reconstructed from the projection stack, using the same projective geometry defined before, resulting in a truncation of around 30% of the patient body and attenuated with the Ohnesorge filter.^35^ Imaging parameters were chosen to match the ones of data set D${}_{r}$, with output CBCT volumes of dimensions 220×220×220 pixels and spacing of 1×1×1 mm. The scatter, beam hardening, and Gaussian noise factors were found empirically and set to 0.001, 1.005, and 0.001, respectively. Conversely to real CBCT, the generated synthetic CBCT resulted perfectly aligned to the corresponding CT, avoiding anatomical deformations and air pockets differences (Figure 2).

### 2.3 Preprocessing

Preprocessing steps were performed before feeding data to the neural network. First, rigid registration was applied, using the ITK API (the Insight ToolKit^38^), only to D${}_{r}$, as D${}_{s}$ was intrinsically already aligned. A masking procedure was then applied to the CT scans of both D${}_{r}$ and D${}_{s}$ data sets to extract the FOV equal to the corresponding CBCT. Every CT volume was resampled to have isotropic voxels of 1×1×1 mm matching CBCT voxel dimensions. The HU range of the grayscale values was first clipped to [−1000,3100] and then rescaled to [0,1] with a linear mapping. Finally, as a technical convenience in the U‐Net processing, the axial slices were zero‐padded to 256×256 pixels from the original size of 220×220 pixels.

### 2.4 Deep convolutional neural network model

**FIGURE 3:**
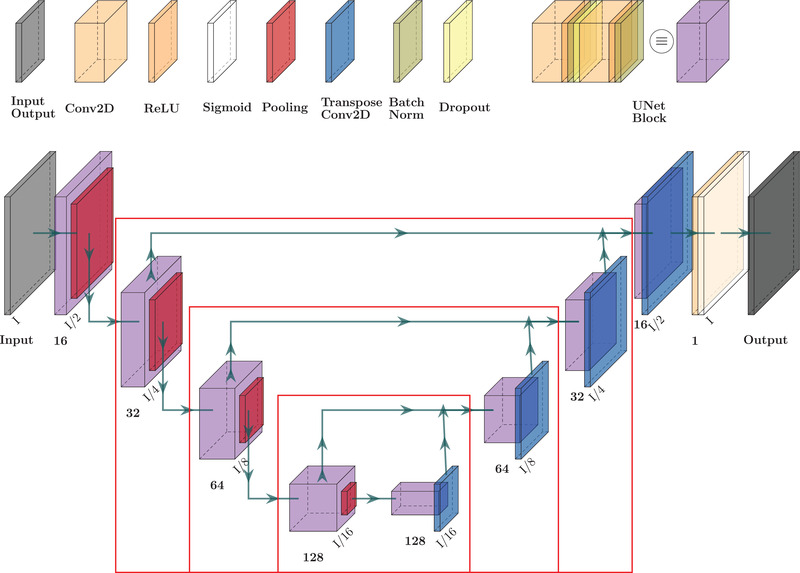
Schematic of the symmetric contracting and expanding paths of the U‐Net. Every U‐Net processing block (light purple) is composed of two convolutional (light orange), Relu (orange), and batch normalization (yellow) layers, with a dropout (light yellow) layer in the middle. Every block in the contracting path is followed by a max‐pooling (red) layer, while every block in the expanding path is followed by a transpose convolution (blue) one. The arrows that link two processing blocks at the same level of both paths indicate a concatenation operator. The last convolution is followed by a Sigmoid (white) layer. The red boxes indicate the blocks that can be retrained during each transfer learning experiment

The basic U‐Net architecture is mainly used for image segmentation,^39^ solving a pixel‐by‐pixel classification problem. In the present work, it was adapted to solve an image‐to‐image translation problem to address the task of cupping removal and HU recovery from the original CBCT. In agreement with the U‐Net architecture, the proposed neural network was mainly composed of a contracting and an expanding path (Figure 3).

In the contracting path, each processing layer (block) was constituted by two 2D convolutions, with kernel dimension 3×3, no stride, and rectified linear unit (ReLU) activation function. A batch normalization layer followed every convolution. Between two convolutions of the same block, a dropout layer randomly “switched off” the updating for 10% of the weights to prevent overfitting. Each block in the contracting path was connected to the next with a max pooling layer, giving as input for the next block a tensor with twice the feature maps and halving its size. The purpose of this contracting path was to capture the context of the input image. The expanding path had the same structure as the contracting path but with a transpose convolution layer instead of max pooling, giving an up‐sampling effect to the network. Thus, each block in the expanding path outputs a tensor with half the feature maps and twice its size. Every block in this path was also connected to its corresponding block in the other paths with a connection layer. The purpose of the expanding path was to enable precise localization combined with contextual information from the contracting path. A single feature map 2D convolution was applied in the last layer, resulting in a single image with the same dimension as the input. The last layer used a sigmoid activation function to provide values in the [0,1] range for every pixel.

### 2.5 Training of the models

Training a U‐Net requires an abundant quantity of data. In the interest of reducing potential overfitting,^40^ data augmentation was performed at runtime during the network training. A series of random operations were applied to the existing data (the input CBCT and its corresponding CT ground‐truth) so that the network was never fed twice with the same image during training. At first, random cropping of the image was performed, feeding the network with a 128×128 subpatch of the original image. These subpatches were also subjected to random rotations of multiples of 90 degrees and horizontal flips. Moreover, input (CBCT) and label (CT) must represent the same anatomical condition. Otherwise, the network will be forced to compensate for residual deformation. The common practice^17^ requires the use of a DIR algorithm to match CT to CBCT daily anatomy as a preprocessing step. This step was avoided on the basis of D${}_{r}$ characteristics (as described in Section 2.2). The synthetic D${}_{s}$ CBCT can be perfectly superimposed on the reference CT from which they were generated, completely avoiding DIR in data preparation (Section 2.2.1).

**FIGURE 4:**
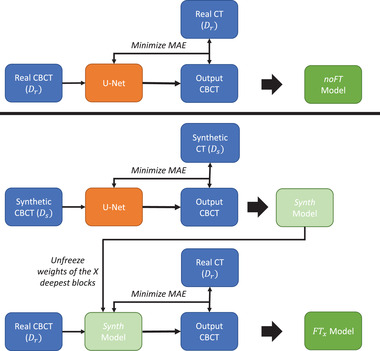
Training pattern for the noFT (upper panel) and FT${}_{x}$ (lower panel) models. The noFT model is trained in a single step using only data from D${}_{r}$. The FT${}_{x}$ model is trained in two steps. In the first one, a model is trained using only D${}_{s}$ (Synth model), then only x (1, 2, or 3) processing blocks were retrained with D${}_{r}$ data

The models analyzed in this work were trained following two different training patterns (cf. Figure 4). The first, named the noFT model, was trained in a single step using only data from D${}_{r}$. The second, named the FT${}_{x}$ model, was trained using both D${}_{s}$ and D${}_{r}$ data sets, following a two‐step approach similar to that of Gherardini et al.^41^ At first, end‐to‐end training was performed using the synthetic data from D${}_{s}$ on a newly created model (the Synth model). In the second step, some deeper processing blocks (one, two, or three) were fine‐tuned using D${}_{r}$, leaving the rest of the model weights fixed. Each retrained block was considered symmetrically in the contracting and expanding paths (cf. Figure 3). Depending on the number of retrained processing blocks, the model takes the name FT${}_{1}$, FT${}_{2}$, FT${}_{3}$. Synthetic data are suitable for augmenting the data set overall dimension and learning the anatomical‐related features in the image. Transfer learning embeds these features in the network, giving a good initialization of the network weights. Therefore, subsequent tuning on real data is supposed to be less sensitive to residual deformation between input and ground‐truth labels.

#### 2.5.1 Loss function and performance metrics

Mean absolute error (MAE) has been set as a loss function, and the training was optimized with ADAM (adaptive moment estimation).^42^ ADAM has the advantage of being designed to compute individual adaptive learning rates for different parameters starting from estimates of first and second moments of the gradients. ADAM optimizer was set with the following parameters: learning rate 0.001, exponential decay rate for the first moment estimates β1=0.9, and exponential decay rate for the second‐moment estimates β2=0.999, while the learning rate was 0.001. To evaluate the network performance, two widely used metrics were chosen, namely, the peak signal‐to‐noise ratio (PSNR) and the structural similarity index measure (SSIM). PSNR value approaches infinity as the mean squared error between improved CBCT and ground‐truth CT approaches zero. Therefore, a higher PSNR value corresponds to higher image quality and vice versa.^43^ SSIM is considered to be more correlated with the quality perception of the human visual system. The higher its value is, the better is the perception of similarity for human eyes.^44^

#### 2.5.2 Hyperparameter tuning experiments

In order to find the optimal U‐Net architecture according to PSNR and SSIM, some experiments were conducted using exclusively D${}_{r}$ data. These experiments aimed to find the best combination for the number of processing blocks (four or five) and the number of feature maps at the first level (16, 32, or 64), systematically varying these parameters. A deeper network with many feature maps can virtually learn more from the input data itself, but it also requires computational power accordingly. The correct trade‐off between these parameters has to be found. All considered architectures were trained using data set D${}_{r}$ and performance results were computed in terms of median and interquartile ranges (IQRs). The statistical difference between the candidate models was evaluated with Kruskal–Wallis nonparametric test for median differences (p< 0.01) and post hoc comparison. If two or more models did not present significant differences, the choice fell on the lighter architecture in terms of computational cost.

#### 2.5.3 Transfer learning experiments

Once the best architecture was chosen according to hyperparameter tuning experiments, some transfer learning experiments were conducted to find the optimal number of processing blocks to be retrained (one, two, or three) to increase PSNR and SSIM. For example, considering the architecture 16‐32‐64‐128‐128‐64‐32‐16 (four processing blocks and sixteen feature maps in the first block), the fine‐tuning of only one block meant that the retraining allowed the refinement of the weights in the 128‐128 level. Conversely, the fine‐tuning of two blocks meant that the retraining allowed the refinement of the weights in the 64‐128‐128‐64 processing blocks. Even in this case, the appropriate number of blocks was chosen by evaluating the statistical differences between experimental results and the computational cost.

#### 2.5.4 Network implementation

The network model, loss function, metrics, and training routine were built using the Keras^45^ and TensorFlow^46^ frameworks in Python. The training was carried out in a Google Colaboratory Cuda‐enabled environment, equipped with a 4‐core CPU, 25 GB RAM, and NVIDIA^®^ Tesla^®^ P100 GPU support 16 GB RAM. The training routine was set to save the best weights values when the validation set SSIM score is maximized.

### 2.6 Cross‐validation analysis

In order to make the network output comparable to the original volumes, the amplitude [0,1] of the network output was scaled back to HU units [−1000,3100]. To reinforce the interpretation of the results, a leave‐one‐out cross‐validation (LOO‐CV) experiment was performed. The Synth model was fine‐tuned with 18 subsets of data set D${}_{r}$, each one excluding a single stack of slices, corresponding to unique CBCT/CT pair P${}_{x}$. A comparison between baseline performance (Base), noFT, and FT${}_{x}$ models was presented to show the validity of the transfer learning approach. Two analyses were conducted to assess: (1) the improvement for PSNR and SSIM (2) the reliability of HU recovery.

#### 2.6.1 Performance metrics

Given the small size of D${}_{r}$ compared to D${}_{s}$, the NoFT model risks overfitting training data. In order to validate the coherency of the performance in terms of PSNR and SSIM with respect to the parameters defined in the previous experiments, LOO‐CV was used to compare the noFT and FT${}_{2}$ model with the baseline (Base) performances. The term Base was used to name the performance computed on the data set D${}_{r}$ as is after rigid alignment and resampling, without any neural network elaboration. The expected result is that the FT${}_{x}$‐type models are more consistent than noFT ones. To verify this assumption, the PSNR and SSIM metrics were computed again on each fold.

#### 2.6.2 HU analysis and shading evaluation

**FIGURE 5:**
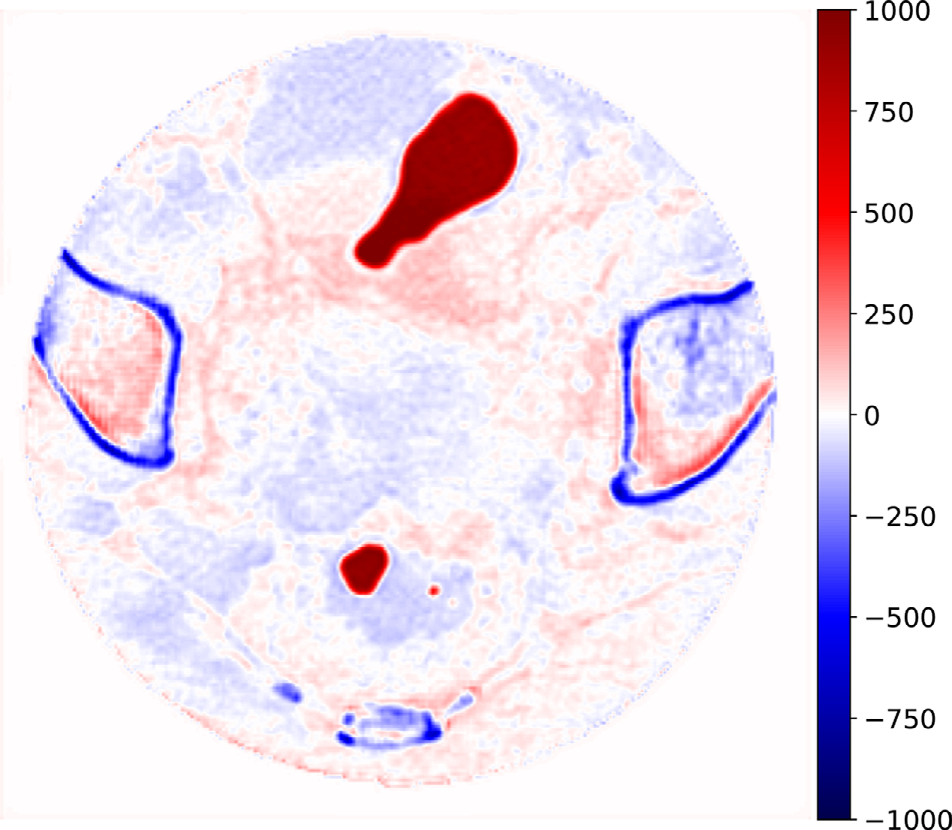
Example of air pockets, visible as a red blob. Since these regions mismatch between the two images, corresponding voxels are not considered for HU difference computation

**FIGURE 6:**
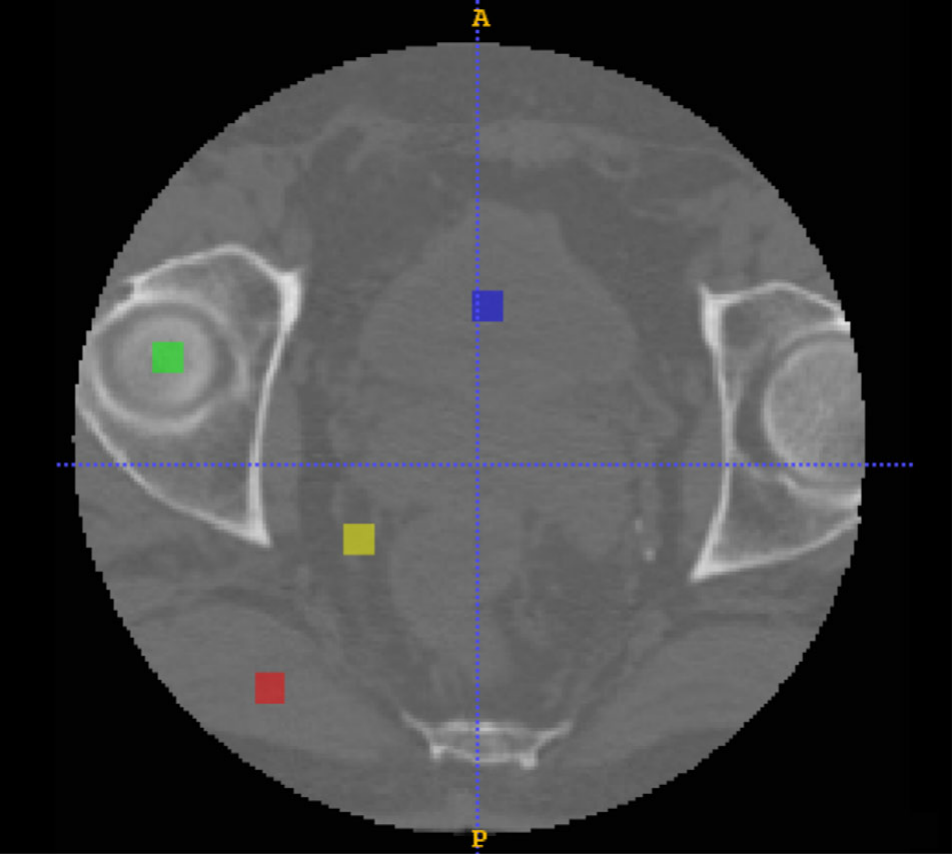
Example of cubic ROIs (8×8×8 mm) extracted from a patient. Selected regions are bladder (blue), spongy bones (green), muscle (red), and fat (yellow)

As far as HU recovery is concerned, the difference in terms of HU between the CBCT network output versus the corresponding CT images was computed. In order to increase the consistency of the comparison, mismatching air pockets were automatically removed according to predefined HU thresholds^1^ (Figure 5). Additionally, an analysis based on region of interest (ROI) was proposed to evaluate the network improvement for different tissues; 8×8×8 cubes were segmented in spongy bones, fat, muscle, and bladder (Figure 6). According to the represented tissue, the HU values contained in each cube were averaged and compared between CT and CBCT. In addition, the contrast‐to‐noise ratio (CNR) was calculated to evaluate the improvement in terms of contrast enhancement. In particular, the CNR was measured for each imaging modality by comparing the CTV with the bladder, muscle, and fat ROIs, respectively. Then, the same ROIs were compared with the air region within each scan. Particular attention was given to the relation between truncation severity due to variable patients pelvic size and HU nonconformity. A detailed description of the analysis can be found in the supporting information (S4).

## 3 RESULTS

### 3.1 Neural network assessment

#### 3.1.1 Hyperparameter tuning experiments

In order to find the best network architecture in terms of PSNR and SSIM, some hyperparameter tuning experiments were conducted (cf. Table 1). In terms of PSNR, all the four‐block architecture provided results significantly better than the five‐block ones (p < 0.0001). Considering four as the number of processing blocks, no statistical difference among 16 and 32 feature maps was found (p = 0.46), as well as between 16 and 64 feature maps (p = 0.74). Therefore, 16 was chosen as it significantly reduces the number of trainable parameters, being the best trade‐off between performance and network complexity. The SSIM analysis further confirmed this choice. The selected U‐Net configuration was 16‐32‐64‐128‐128‐64‐32‐16, corresponding to 919177 trainable parameters.

**TABLE 1:** PSNR and SSIM performances (median and interquartile range) of the hyperparameter tuning experiments, depending on the number of processing blocks and the number of convolutional filters in the first block. Each value is computed evaluating the data set D${}_{r}$ test set. The final choice for these parameters for both noFT and FT${}_{x}$ models is in bold

Blocks #	First block filter #	PSNR (dB)	SSIM (A.U.)
**4**	**16**	**31.943 (3.261)**	**0.926 (0.030)**
4	32	32.156 (2.913)	0.918 (0.025)
4	64	32.314 (2.661)	0.925 (0.028)
5	16	31.704 (2.674)	0.928 (0.029)
5	32	31.608 (2.503)	0.918 (0.023)
5	64	30.367 (2.274)	0.926 (0.027)

#### 3.1.2 Transfer learning experiments

Considering the transfer learning experiments results (cfr. Table 2), retraining the two deepest blocks proved to be the best value for increasing network performance. This was confirmed by the statistical analysis of the FT${}_{1}$, FT${}_{2}$, and FT${}_{3}$, considering PSNR, SSIM, and MAE. For PSNR, FT${}_{2}$ architecture provided results significantly better than the other two FT${}_{1}$ (p < 0.0001) and FT${}_{3}$ (p< 0.0001) architectures. Considering SSIM, no statistical difference among FT${}_{1}$ and FT${}_{3}$ (p = 0.78) was found, while the FT${}_{2}$ results to be significantly different from FT${}_{1}$ (p= 0.007) and FT${}_{3}$ (p= 0.0007). In addition, the three distributions were compared with the distribution obtained from the Synth model. In general, each FT${}_{x}$ model had significantly better performance compared to the Synth model, both in terms of PSNR (p < 0.0001), SSIM (p < 0.0001), and MAE (p < 0.0001). As such, Synth was not considered for further evaluations.

**TABLE 2:** PSNR, SSIM, and MAE performances (median and interquartile range) of the transfer learning experiments, depending on the number of processing blocks to be retrained. Each value is computed evaluating the data set D${}_{r}$ test set. The final choice for the FT${}_{x}$ model (FT${}_{2}$) is in bold

FT blocks #	PSNR (dB)	SSIM (A.U.)	MAE (HU)
0 (Synth)	26.707 (1.440)	0.907 (0.018)	128.184 (28.011)
1	29.007 (2.151)	0.918 (0.024)	84.989 (23.187)
**2**	**30.799 (2.167)**	**0.921 (0.023)**	**63.067 (15.179)**
3	29.904 (2.268)	0.917 (0.025)	73.187 (19.726)

### 3.2 Cross‐validation analysis

#### 3.2.1 Performance metrics

**FIGURE 7:**
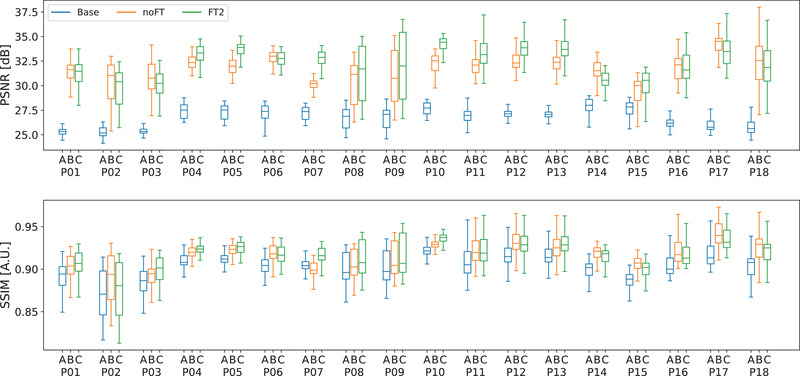
Quantitative analysis of PSNR and SSIM values between every CBCT (Base, noFT, FT${}_{2}$), computed for each fold of the leave‐one‐out cross‐validation

**FIGURE 8:**
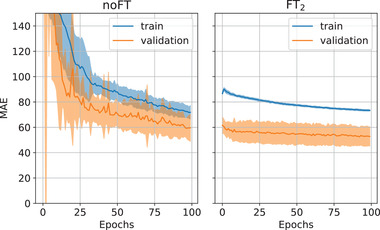
Mean absolute error history for noFT (left) and FT${}_{2}$ (right) models during training. Bold lines represent the mean values computed between each trained network in LOO‐CV experiments, while the shaded region represents their standard deviation

The LOO‐CV experiments confirmed that the proposed neural network processing improved the quantification metrics with respect to baseline (Figure 7). On most of the patients, the FT${}_{2}$ model performed better than the Base and noFT in terms of PSNR and SSIM. In particular, the median PSNR for Base, noFT, and FT${}_{2}$ were on average, over the 18‐fold, 26.77, 31.83, and 32.32 dB, respectively. Considering PSNR, FT${}_{2}$ model results to be significantly better than Base (p< 0.0001) and noFT (p < 0.0001). The median SSIM for Base, noFT and FT${}_{2}$ were on average 0.902, 0.915, and 0.916. Even in this case, FT${}_{2}$ model results to be significantly better than Base (p < 0.0001) and noFT (p = 0.0005). Furthermore, by aggregating the training histories of the 18 LOO‐CV experiments for both models and comparing the MAE for the training and validation sets, the FT${}_{2}$ model proved to be faster in terms of convergence speed (cf. Figure 8). This is due to the FT${}_{2}$ network weights initialization provided by the pretraining with synthetic data.

#### 3.2.2 HU analysis and shading evaluation

**FIGURE 9:**
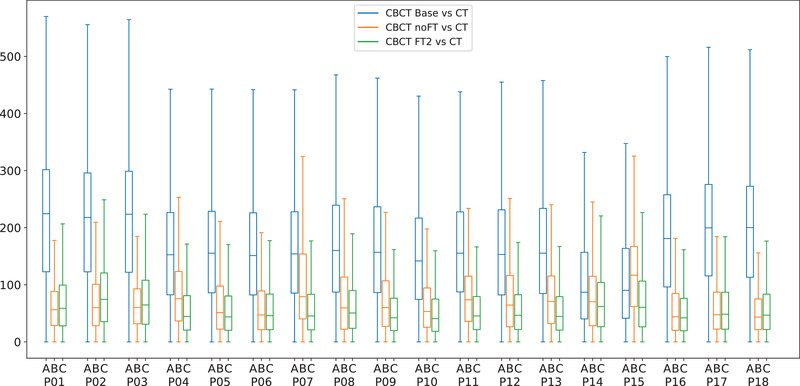
Quantitative analysis of the absolute HU difference between every CBCT (Base, noFT, FT${}_{2}$) and the corresponding ground‐truth CT, computed for each fold of the leave‐one‐out cross‐validation. Both models reduce the difference in the HU ranges, with better performance for the FT${}_{2}$ model.

The ROI evaluation produced, on average, HU improvements of 177.59 for bladder, 192.2 for bone, 215.87 for muscle and 123.43 for fat using the noFT method. By contrast, the FT${}_{2}$ method yielded an average HU improvement of 191.95 for bladder, 257.04 for bone, 206.88 for muscle and 169.73 for fat. For the whole images (Figure 9), the FT${}_{2}$ method obtained a median improvement of 111.96, while the noFT yielded 100.52. For each ROI, the comparison between noFT and FT${}_{2}$ HU difference distribution reported a significative difference (p < 0.0001). All values were computed across the 18‐fold and are summarized in Table 3.

**TABLE 3:** Absolute HU difference between every CBCT (Base, noFT, FT${}_{2}$) and the corresponding ground‐truth CT, for each ROI (mean and standard deviation) and overall volumes (median and interquartile range). Values are obtained averaging between each fold of the leave‐one‐out cross‐validation

Model	Bladder	Bone	Muscle	Fat	All
Base	213.06 ±47.19	340.79±66.06	249.36±20.53	199.27 ±39.46	161.37 (162.54)
noFT	35.47±25.27	148.59±74.25	33.49±28.78	75.84 ±28.02	60.85 (80.70)
FT${}_{2}$	23.11±20.87	83.75±55.41	42.48 ±28.83	29.54±19.40	49.41 (66.70)

**FIGURE 10:**
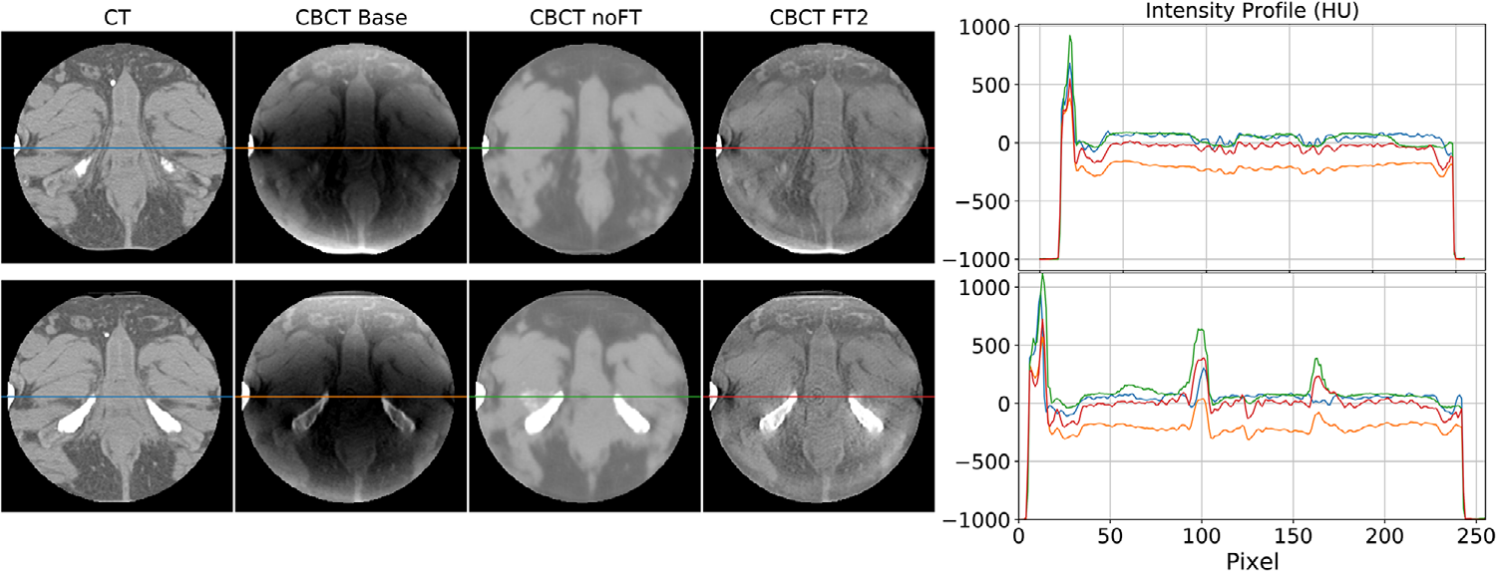
Comparison between a single CBCT Base and corresponding CT axial slice with the CBCT elaborated by noFT and FT${}_{2}$ models. The rightmost part of the figure compares the intensity profiles of the central line of the images, highlighted by the central line in the four representations. Images are displayed with Window = 400, Level = 20

The cupping artifact, due to scattering, clearly perceived as a darker central region in CBCT${}_{Base}$, was reduced in both CBCT${}_{noFT}$ and CBCT${}_{FT_{2}}$ (Figure 10). Additional examples with a better resolution can be seen in the supporting information (S3), also showing ring artifacts in all CBCT scans caused by suboptimal flat‐panel calibration, to a different degree of severity. The analysis of one intensity profile (the central row depicted with a line in Figure 10) confirmed that the network processing flattened the slight concavity present in CBCT${}_{Base}$. The intensity profile of CBCT${}_{noFT}$ appeared closer to the CT one than the corresponding profile obtained from CBCT${}_{FT_{2}}$. At the same time, CBCT${}_{noFT}$ profiles appear smoother than the CT one and its axial view is blurred. This evidence supports the quantitative results for HU in ROIs and overall image, with FT${}_{2}$ performing better than noFT. It follows that the FT${}_{2}$ and the noFT method both shift the range of values of CBCT closer to that of CT. The FT${}_{2}$ model favored anatomical consistency, correcting cupping, and rescaling the intensity values. Conversely, the noFT model aggressively fits the CBCT to the reference CT, introducing blurring.

The results of the tissue contrast analyses are summarized in Table 4. In all cases, the FT${}_{2}$ model obtained results closest to the CT ones, considered the ground‐truth. These results confirmed the superior ability of this model in improving soft tissue visibility with respect to CBCT${}_{Base}$ or CBCT${}_{noFT}$. In particular, the comparison between CTV and soft tissues reported an improvement of about 24% for the noFT model and 67% for the FT${}_{2}$ one, with respect to the CBCT${}_{Base}$ CNR values of CBCT${}_{Base}$. Concerning the comparison between soft tissues and air, the average gain was about 4% and 35% for noFT and FT${}_{2}$, respectively.

**TABLE 4:** CNR values for every imaging modalities (CT, CBCT${}_{Base}$, CBCT${}_{noFT}$, CBCT${}_{FT_{2}}$). Values are computed between clinical target volume against every soft tissue ROI (bladder, muscle, fat). Every ROI is also evaluated against air values present in the scan. Each value is represented as median (IQR)

ROI					
Foreground	Background	CT	CBCT${}_{Base}$	CBCT${}_{noFT}$	CBCT${}_{FT_{2}}$
CTV	Bladder	0.86 (1.00)	−5.24 (2.28)	−1.73 (2.35)	−1.19 (3.31)
	Muscle	−1.75 (1.76)	−5.12 (2.01)	−5.68 (6.27)	−1.66 (2.31)
	Fat	4.68 (1.13)	1.11 (2.31)	2.19 (3.19)	2.49 (2.08)
Bladder	Air	42.06 (15.23)	7.79 (2.93)	8.66 (3.25)	19.47 (14.05)
Muscle		43.59 (15.76)	7.69 (2.51)	8.86 (2.63)	19.28 (13.49)
Fat		37.57 (13.29)	6.82 (3.29)	8.23 (3.02)	18.69 (12.80)
CTV		42.48 (18.26)	7.08 (2.90)	8.70 (2.96)	19.42 (13.45)

**FIGURE 11:**
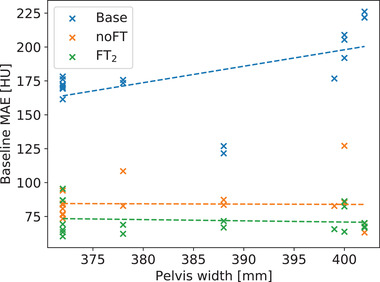
Pelvis width versus MAE for every type of CBCT (Base, noFT, FT${}_{2}$). MAE is calculated with respect to the CT ground‐truth

Results regarding the relationship between MAE and pelvis width for considered cases are reported in Figure 11.

## 4 DISCUSSION

In this work, we propose a U‐Net–based approach to address limitations intrinsic to the narrow‐FOV CNAO CBCT, and to provide CBCT HU recovery, subsequently. In particular, the framework proposed focused on shading correction and soft tissue contrast enhancement. First, the network improved the image intensity distribution, quantified by PSNR and SSIM, in the range of about 5 dB (noFT), 6 dB (FT${}_{2}$), and 2% (both), respectively. These highlight global contrast and signal‐to‐noise ratio improvement. The relative improvement of CNR for the CTV versus various soft tissues was on average 24% (noFT) and 67% (FT${}_{2}$). Coherently, the average CNR improvement for soft tissue with respect to the air in the CBCT scans was 4% (noFT) and 35% (FT${}_{2}$). Second, the network was able to provide HU grayscale values comparable to the ground‐truth CT, reducing the nonlinear cupping artifact and scaling the intensity values. In addition, MAE results for noFT and especially for FT${}_{2}$ models improved both in median (62.29% and 69.38%, respectively) and narrower IQR (50.35% and 58.96%, respectively) terms. The latter indicates a compensation and generalization capability of the two approaches with respect to different pelvis widths and, therefore, truncation, as shown by Figure 11. Finally, both networks performed the required task. Performances were similar, but the HU comparison for ROI and the overall image showed a performance edge of the FT${}_{2}$ model. Consequently, preconditioning the network with synthetic data proved to be an effective method for the problems addressed in this work. The two‐step training allowed to split the learning of the anatomical features from the learning of CBCT/CT shading differences.

Existing literature using U‐Net architecture aims at correcting CBCT with sufficient FOV^17^, ^18^, ^19^, ^25^, ^26^ and generally does not encompass truncation artifacts, except for one work exploiting MC simulations on synthetic data.^11^ Our work evaluates a similar deep learning architecture tailored to CNAO needs. The aforementioned works validate their approach by means of dosimetric accuracy. The proposed methods are tested on the overall geometrical improvement in the image HU. In order to further evaluate possible clinical applications, a dosimetric study will be eventually paired to the HU analysis hereby presented. However, achieving dose calculation on narrow‐FOV CBCT scans is currently out of the scope of this work. Comparing the HU difference between CT and CBCT with existing methods leveraging CT information, while most of these works do not address truncated data, we can assess the proposed network performance. For instance, using an MC‐based methodology, Thing et al. reported HU correction of about 31% for five lung cancer patient images.^7^ Another method based on histogram matching on ten prostate cancer patient images provided HU correction of about 20%.^47^ Phantom studies have reached up to a 95% overall accuracy in the HU recovery on the Catphan 600^48^ alone, but no data were provided for patients. In comparison, our FT${}_{2}$ and noFT models respectively obtained around 69.38% and 62.29% average improvement on the whole image in the pelvic region. Addressing the HU correction on different tissues, the improvement for FT${}_{2}$ (noFT) can be described as 75% (56%) in the spongy bone (femoral head), 89% (83%) in bladder, 85% (62%) for fat, and 83% (86%) for muscle. In particular, Kida et al.^17^ investigated a U‐Net approach similar to noFT with wide untruncated FOV, where authors reported improvements of 95% and 94% on the last two tissues. In another work, the authors obtained an improvement of about 90% in terms of HU accuracy. This value was computed with respect to corresponding MC‐corrected CBCT scans used as the ground‐truth reference.^25^ All data in this study had wide FOV, avoiding truncation. Finally, another work using MC simulations to train a CNN on synthetic CBCT scans has obtained great adherence with the MC ground‐truth with as low as 1.4% and 1.8% discrepancies independently of simulated truncation.^11^ This work seems particularly promising on synthetic data, but to compare it properly to our work we would need to reimplement the same architecture, training, and accurate MC simulation for our data set. This is out of the scope for the current article but could be the basis of another work in the future. Specifically, we believe that transfer learning could provide benefits to literature CNN methods as well. Concerning the contrast‐to‐noise improvement, one work proposed a solution based on a deep convolutional autoencoder that gained an improvement of about 42% in terms of CNR, computed evaluating muscle and fat regions from retrospective CBCT scans.^27^ Overall, on real data, the obtained results in the present work are in line with existing methods not accounting for truncation. Moreover, the exclusive use of data set D${}_{r}$ has some limitations. First, this data set has a relatively small size for a deep learning approach, with the risk of overfitting on this set of patients without generalization capability. Second, although the acquisition of CT/CBCT pairs is close in time, the pelvic district involves differences that are not always negligible between the two scans, like air bubbles in the bowel. These problems lead to a suboptimal selection of CT/CBCT pairs and can have a nonnegligible impact on the model performance even when residual deformation itself is as negligible as in our data set. In order to overcome these issues, we performed a two‐step training on FT${}_{2}$ following the transfer learning approach, which increments the overall data set dimensionality while also integrating exact anatomical information:

1. Using synthetically generated CBCT as input, generated from a publicly available CT data set, the network was trained based on perfect anatomical correspondences with ground‐truth CT. This step provided a suitable initialization of the image‐to‐image translation process and allowed mainly to learn the image filters and geometric features, which identify the anatomical district of interest;
2. Using the real CBCT images provided by CNAO, which were only rigidly registered to the corresponding CT scans, the network was partially retrained according to the transfer learning paradigm. In this phase, the training focused on the weights of the inner layers, potentially devoted to learning more complex characteristics related to the intrinsic quality of the image acquired with the specific technology available at the CNAO.

The action of the models (noFT and FT${}_{2}$) can be qualitatively noted by observing the effects they have on the output images (cf. Figure 10). The noFT model aggressively fits the CBCT to the reference CT. In particular, it applies a transformation similar to an averaging filter. Instead, the FT${}_{2}$ model outputs sharper images. The comparison of HU differences on individual ROIs shows coherent results. FT${}_{2}$ model, with respect to the noFT model, achieves a greater improvement of 6% on the bladder, 19% on bone, 23% on fat, and in general 7% on the whole image, while underperforming only by 3% on muscle. Synthesizing, transfer learning proved to be effective for our application. However, it is important to identify the appropriate number of layers to retrain. If this number is too high (or too low), the network performance will be negatively affected. Tuning this value, we assess the correct balance between generic features like shapes, better learned from synthetic data, and artifacts better learned by real‐world clinical data. In this study, we found that the optimal number of layers for retraining is two. Due to the slice‐based training, artifacts are suppressed globally and without disentangling them.

Though the results above reported were promising on clinical data, they are still preliminary. The scope of this work is, by all means, a feasibility study. We provided a shading correction method for a limited FOV CBCT scan data set containing few patients. In order to extend the aim of this work, the data set must be enlarged and a dosimetric analysis has to be associated. Another limited aspect of our work is the network ability to generalize. Our data sets were tied to the pelvis, therefore we cannot assess the impact on other large districts (e.g., lungs). In particular, variation in the patient width is correlated to resulting HU inaccuracies in the input data. However, this impact is attenuated by our network, as briefly described by supporting information (S4). Admittedly, CBCT scans fed to the FT${}_{2}$ network are corrected for shades but not for ring artifacts. These panel‐calibration–induced ripples are already present in the original CBCT volumes, somehow hidden by the more prominent cupping artifact. We believe the noFT model partially compensates for it through its low pass filtering. Since those artifacts are caused by suboptimal flat‐panel calibration, this should not penalize the FT${}_{2}$ model with respect to the noFT approach. In particular, specializing the FT${}_{2}$ model to correct this hardware issue may hinder the generalization of the method. Or rather, we suggest that this condition would be easily solved by properly calibrating the panel rather than retrospectively correcting for the subsequent artifacts. The main unaddressed limitation resides in the missing information due to the narrow FOV of these CBCT scans. The proposed methodology does not try to provide this information alone, but further studies must address this issue. Finally, while results were promising on CNAO data set D${}_{r}$, they are based on the assumption that the residual deformation between input and label is negligible (as discussed in the supporting information [S2]). We hypothesize that the transfer learning approach would be more robust to residual deformation, but this aspect needs further investigation.

The system implemented by Fattori et al.^2^ at CNAO facility was originally intended for accurate X‐ray setup verification. The CBCT provided by the system is currently used for qualitative evaluations and dictates the request for a so‐called reevaluation CT.^9^, ^10^ Improving the images acquired through this instrument has two consecutive advantages. First, a CBCT without shadows and with improved visibility reduces the risk for setup errors by clinicians. Therefore, CBCT scans could be used in lieu of the traditional 2D to 3D X‐ray setup verification. Second, better contrast between CTV and other soft tissues is indicative of more direct visual discrimination. The CNR improvement of those tissues versus air, paired with enhanced HU adherence to CT, supports the use of CBCT scans for air cavities identification in the pelvis. Consequently, the offline clinical procedure will be more efficient and less prone to overestimation and underestimation of air abundance in the bowels. Outside CNAO clinical practice, the use of a public data set for FT${}_{2}$ improves the repeatability of our study. Moreover, the minimum required numerosity for the clinical data set used for training is arguably lower than similar deep learning approaches we found in the literature. Another element that may appeal to the potential clinical application is the lack of additional equipment requirements, as this would not change the already established clinical routine or require additional costs. Moreover, the proposed method has negligible execution runtime compared to image reconstruction, avoiding a bottleneck for clinical practice. By reducing artifacts hindering the HU calibration curve, we obtained a coherent density representation. A volumetric image with these characteristics could establish a base for online adaptive radiation therapy techniques at CNAO. Additionally, we argue that a VirtualCT‐oriented DIR on such corrected data would converge more easily and faster. In conclusion, we foresee studies that leverage this method to study both the corrected CBCT and aforementioned VirtualCT approach with FOV extension^49^ for a dose evaluation on daily anatomy.

## 5 CONCLUSION

Correcting cupping and shading in pelvis CBCT using a U‐Net proved flexible enough to adapt to a data set flawed by truncation artifacts. Moreover, our push to reduce prior knowledge in the network training was successful thanks to transfer learning. We demonstrated that recovering CT‐compatible data from narrow‐FOV CBCT scans with shading artifacts is possible and can be achieved as a quick postprocessing step. Further investigation on clinical usage for the corrected images will ensue with expert evaluation on a larger patient cohort.

## CONFLICT OF INTEREST

The authors have no relevant conflicts of interest to disclose.

## Supporting information

Supporting Information

## Data Availability

The data that support the findings of this study are available on request from the corresponding author. The data are not publicly available due to privacy or ethical restrictions.

## Footnotes

1
https://www.cancerimagingarchive.net
