# Supplementary material for: Image‐based shading correction for narrow‐FOV truncated pelvic CBCT with deep convolutional neural networks and transfer learning
Source: Med Phys. 2021 Oct 26;48(11):7112–26. doi: 10.1002/mp.15282 (PMC9297981; doi:10.1002/mp.15282)
Supplement: Supplementary file 1 — Supporting Information [file MP-48-7112-s001.pdf]

SUPPLEMENTARY MATERIAL

S1. PATIENT DESCRIPTION

TABLE 1

Patient	Sex	CBCT/CT Pair	# Axial Slices	Distance between CT and CBCT (day)	**Pelvis width (mm)**
Patient 1	F	P01	137	0	402
		P02	180	0	
		P03	171	0	
Patient 2	M	P04	195	1	372
		P05	195	1	
		P06	195	1	
		P07	195	1	
		P08	215	0	
		P09	215	0	
		P10	204	0	
Patient 3	F	P11	218	0	399
Patient 4	M	P12	206	0	378
		P13	204	0	
Patient 5*	F	P14	126	2	388
		P15	107	0	
Patient 6	M	P16	188	0	400
		P17	217	0	
		P18	200	0	

* This patient has a metal screw in the spine after surgical operation. Her values are considered outliers in cross-validation analyses.

S2. DEFORMABLE IMAGE REGISTRATION

The clinical dataset analyzed in the main article comprises same-patients CBCT/CT couples that were picked because acquired inside a two-day window. In particular, CT scans used in our dataset are, in fact, re-evaluation CT scans.

CNAO clinical practice requires using thermoplastic masks (Renfu Medical Equipment, Guangzhou, China) for patient alignment, immobilization and internal motion reduction. Such mask is patient-specific, and it is molded to compress the targeted anatomical region. The mask is used at each step of treatment. During planning CT acquisition, CBCT imaging before dose delivery and re-evaluation CT scan acquisition. Therefore, corresponding CBCT/CT should show negligible inter-scan deformation.

In order to properly evaluate this assumption, we tested a Deformable Image Registration (DIR) algorithm implemented in Plastimatch (http://plastimatch.org) on the described couples. Considering the low contrast and limited FOV of the CBCT in our dataset, we chose to use a landmark-based registration in order to achieve better convergence. We placed 14 landmark couples on each of the 18 CBCT/CT cases considered. We choose BSpline optimized by L-BFGSB over Mutual Information. Resolution starts at 8x8x8 mm with grid spacing at 20x20x20 mm, ending at last stage with 1x1x1 mm and 5x5x5 grid spacing.

A summary of the estimated deformation is displayed in Fig.1. Table 2 shows median(IQR) values for each considered case. The estimated median Deformable Vector Field (DVF) length is always under the original CT limit of resolution (2mm). Based on this observation, we considered the assumption of negligible inter-scan deformation to hold.

Finally, we conducted a qualitative comparison of the overlay between CBCT and corresponding CT scans before and after warping according to the estimated DVF. We show two example of DIR outcome: one with a proper compensation (Fig. 2) and the other with an under-estimated DIR result (Fig. 3). This suggests that in some cases DIR algorithm did not fully compensate for motion. We argue that this is mainly due to the poor contrast exhibited by the CBCT scans, which prevent DIR optimization to converge. Nevertheless, we hypothesized that this residual deformation would not influence the proposed method, especially in the case of data augmentation through the transfer learning approach.

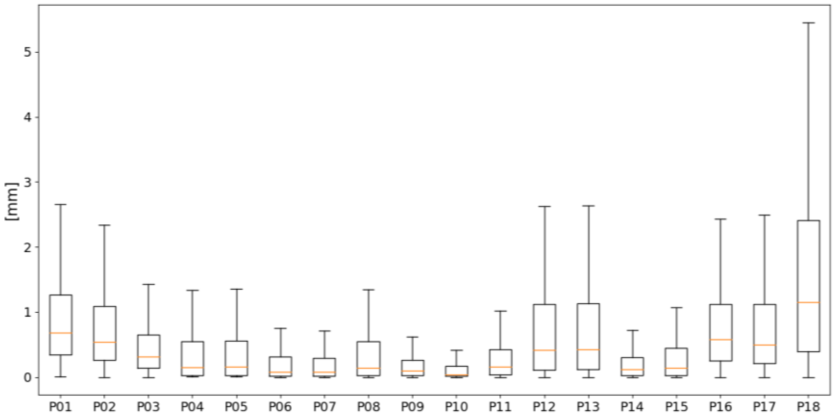

FIG. 1 VECTOR LENGTH IN THE ESTIMATED DEFORMABLE VECTOR FIELD

Case	*Median(IQR) [mm]*
P01	0.69(0.92)
P02	0.54(0.83)
P03	0.32(0.52)
P04	0.15(0.52)
P05	0.16(0.53)
P06	0.08(0.30)
P07	0.07(0.28)
P08	0.14(0.53)
P09	0.10(0.24)
P10	0.04(0.16)
P11	0.16(0.39)
P12	0.41(1.00)
P13	0.42(1.01)
P14	0.12(0.27)
P15	0.14(0.42)
P16	0.58(0.87)
P17	0.50(0.91)
P18	1.15(2.02)

TABLE 2

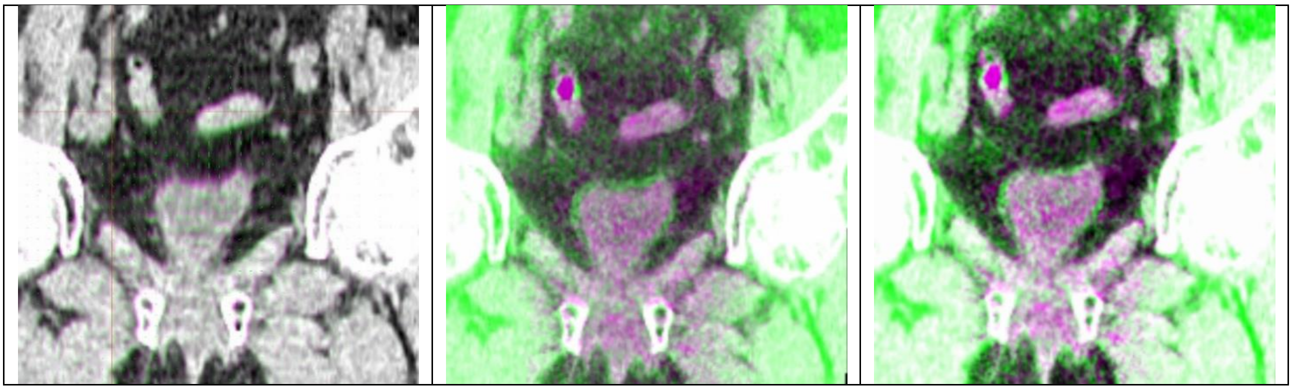

FIG. 2 P11 CT VS CTWARPED (LEFT), CBCT VS CT ORIGINAL (CENTER), CBCT VS CT WARPED (RIGHT)

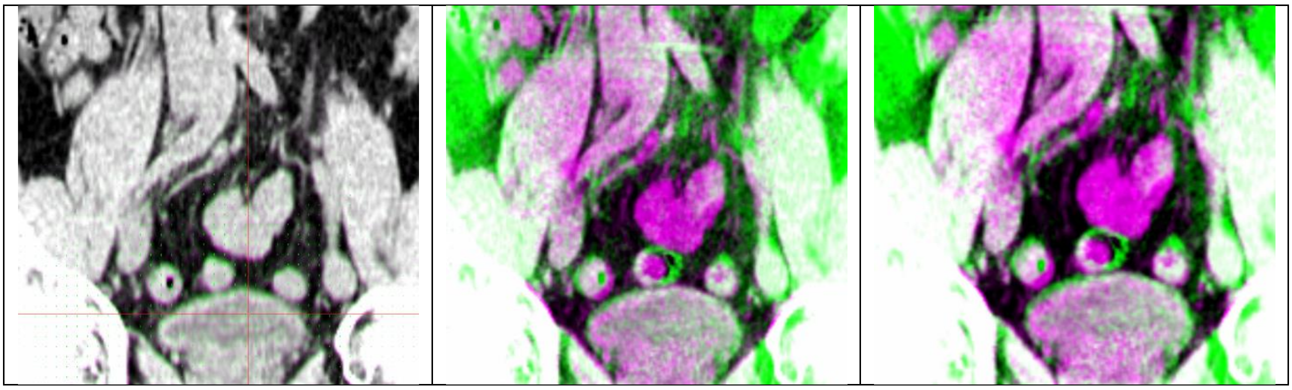

FIG. 3 P05 CT VS CTWARPED (LEFT), CBCT VS CT ORIGINAL (CENTER), CBCT VS CT WARPED (RIGHT)

S3. EXAMPLES OF COMPARISON BETWEEN CORRESPONDING AXIAL SLICES

Every image is displayed with Window=400 and Level=20.

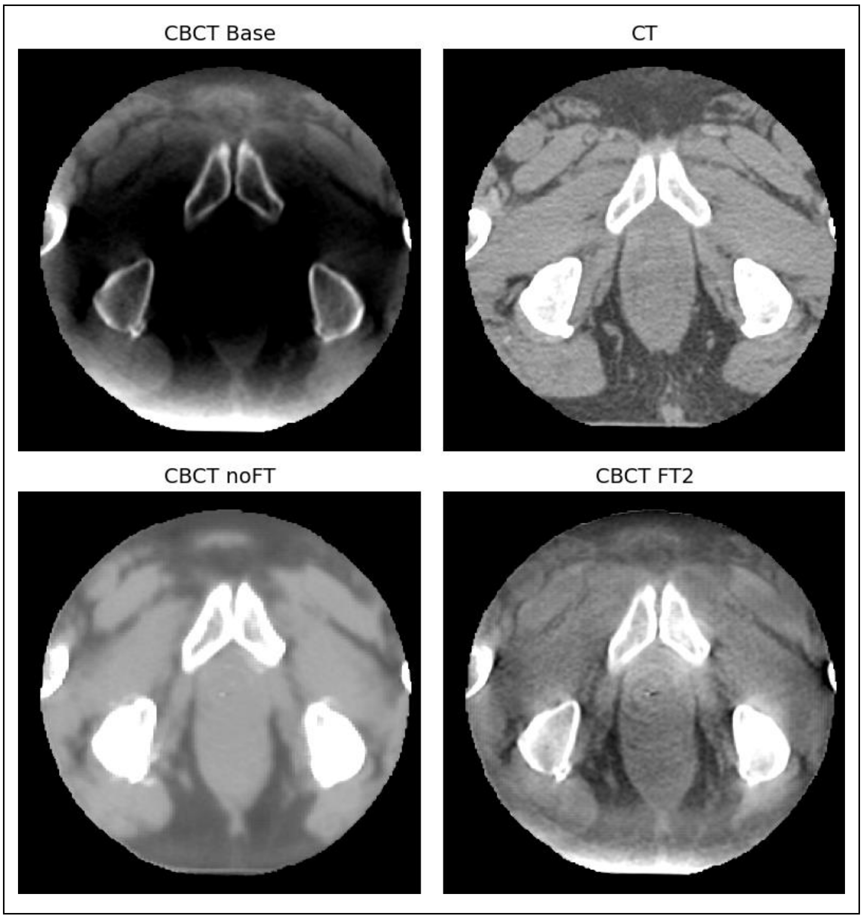

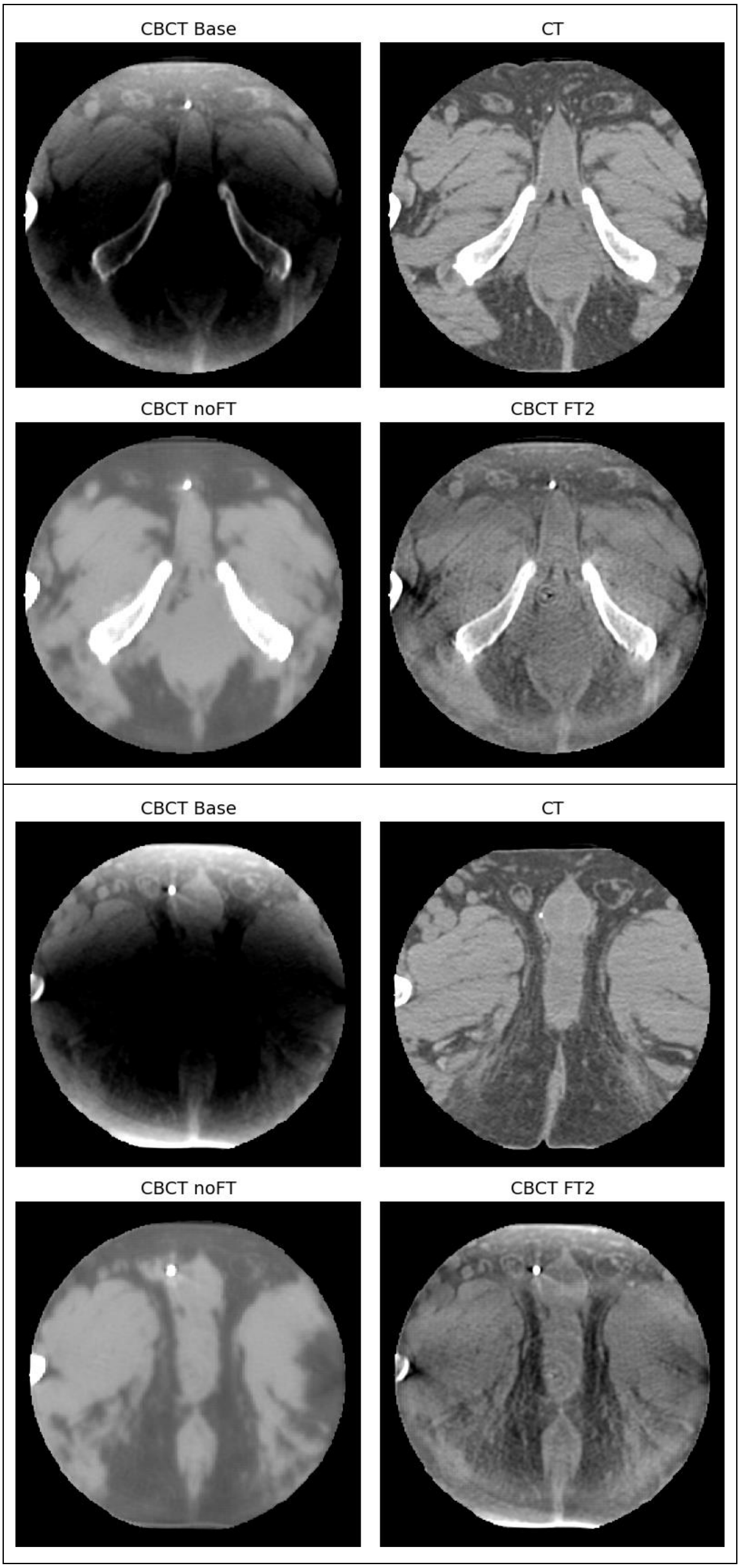
CBCT Base
CT
CBCT noFT
CBCT FT2
CBCT Base
CT
CBCT noFT
CBCT FT2

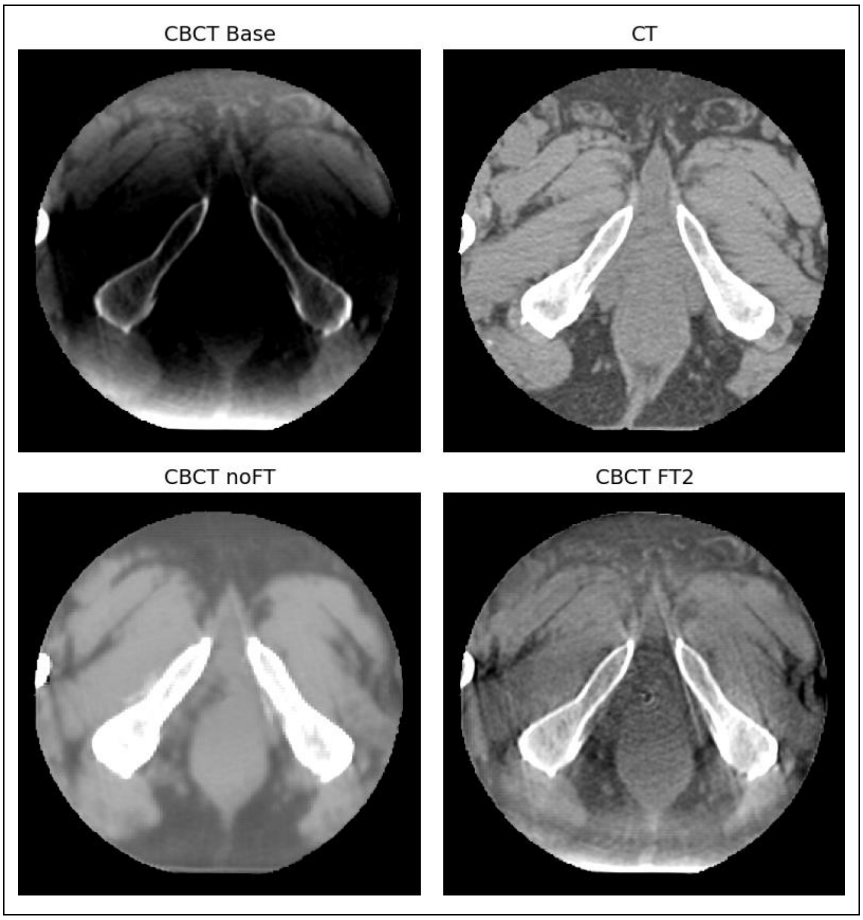

FIG. 4 EXAMPLES OF COMPARISON BETWEEN CORRESPONDING AXIAL SLICES IN EVERY MODALITY (CBCT BASE, NOFT, FT2 AND CT)

S4. RELATIONSHIP BETWEEN PELVIS WIDTH AND CBCT BASE MAE

Table 3 reports the size of each patient's pelvis in ascending order, along with the corresponding mean absolute error (MAE) for every type of CBCT (baseline, noFT, FT_2). MAE is calculated with respect to the CT ground truth. Every CBCT scan is acquired with a FOV of 208 mm and processed by a 30\% truncation correction with Ohnesorge filter. This value is adopted by CNAO clinical practice as a constant. Therefore, in order to maintain this study compliant with clinical routines, the percentage of truncation correction was not modified according to the size of the specific subject. This is reflected in a more significant distortion of the image when patient size increased, visible as a growing MAE for baseline CBCT (Figure 5, blue line). As previously stated in Table 1, Patient 5 (P14 and P15 pair) was considered an outlier for this analysis since this subject had a metal screw in the spine after surgical operation. This condition was no longer present after CNN processing, as demonstrated by the flat line corresponding to noFT and FT_2 cases (Figure 5, grey and orange line).

TABLE 3

Patient	*CBCT/CT Pair*	*Pelvis width (mm)*	*MAE Base (HU)*	*MAE noFT (HU)*	*MAE FT2 (HU)*
Patient 2	P10	372	161.43	84.53	95.47
Patient 2	P04	372	169.24	80.78	86.99
Patient 2	P06	372	170.03	83.67	86.83
Patient 2	P05	372	171.41	76.88	60.33
Patient 2	P07	372	172.37	94.13	65.20

Patient 2	P09	372	176.17	73.39	69.30
Patient 2	P08	372	178.24	76.26	63.42
Patient 4	P12	378	173.46	108.42	68.86
Patient 4	P13	378	175.72	82.82	62.21
Patient 5	P14	388	121.57	83.54	71.65
Patient 5	P15	388	126.91	87.31	66.85
Patient 3	P11	399	176.75	82.76	65.62
Patient 6	P16	400	191.92	86.15	63.74
Patient 6	P18	400	205.37	85.34	82.40
Patient 6	P17	400	208.93	127.11	86.18
Patient 1	P02	402	221.69	69.98	66.77
Patient 1	P03	402	221.76	63.17	67.55
Patient 1	P01	402	226.09	70.00	70.20
